# Clinical utility of copeptin in histiocytosis-associated arginine vasopressin deficiency: a report of three cases

**DOI:** 10.1007/s00277-026-07179-7

**Published:** 2026-07-15

**Authors:** Luca Malatesta, Giuliana Marongiu, Francesco Pegoraro, Stefano Stagi, Marzia Mortilla, Marco Allinovi, Anna M. Buccoliero, Elena Sieni, Augusto Vaglio

**Affiliations:** 1 Department of Biomedical Experimental and Clinical Sciences “Mario Serio”, University of Florence, Viale Pieraccini 6, Florence, Italy; 2 Haematology and Oncology Department, Meyer Children’s Hospital IRCCS, Firenze, Italy; 3 Department of Experimental and Clinical Medicine, University of Florence, Florence, Italy; 4 Department of Health Sciences, University of Florence, Florence, Italy; 5 Auxo-endocrinology Division, Meyer Children’s Hospital IRCCS, Florence, Italy; 6 Neuroradiology, Meyer Children’s Hospital IRCCS, Florence, Italy; 7 Nephrology, Dialysis and Transplantation Unit, Careggi University Hospital, Florence, Italy; 8 Pathology Unit, Meyer Children’s Hospital IRCCS, Florence, Italy; 9 Pediatric Hematology- Oncology Department, Meyer Children’s Hospital IRCCS, Florence, Italy; 10 Nephrology and Dialysis Unit, Meyer Children’s Hospital IRCCS, Florence, Italy

**Keywords:** Langerhans-cell histiocytosis, Rosai-Dorfman disease, Erdheim-Chester disease, copeptin, arginine vasopressin deficiency.

## Abstract

Arginine vasopressin deficiency (AVP-D) is a frequent complication of histiocytosis and may precede the systemic manifestations of the disease of several years. Currently, no reliable biomarkers allow the diagnosis of AVP-D in the setting of histiocytosis. Traditional diagnostic methods, such as the water deprivation test, are often impractical. Copeptin, a new surrogate marker for arginine vasopressin, has been used for the diagnosis of AVP-D in other conditions, such as trauma and malignancies. We herein report three clinical cases of systemic histiocytosis (one Erdheim-Chester disease, one Rosai-Dorfman-Destombes disease, and one Langerhans cell histiocytosis) where plasma copeptin was utilized to identify AVP-D. The three cases differed not only because of the underlying diagnosis, but also in terms of clinical characteristics and rapidity of onset of AVP-D. We propose an early integration of copeptin into the diagnostic workup of systemic histiocytosis.

## Introduction

Arginine vasopressin deficiency (AVP-D), historically known as central diabetes insipidus, is characterized by the excretion of large volumes of hypotonic urine and should be suspected in the presence of polyuria, low urine osmolality, high plasma osmolality, hypernatremia, hyposthenuria, and dehydration; if not promptly identified, AVP-D can lead to severe and potentially life-threatening complications. The underlying cause may be a deficient production or secretion of arginine-vasopressin (AVP) by the pituitary gland/hypothalamus, or a resistance to AVP in the kidney (nephrogenic diabetes insipidus) [1]. The main causes of AVP-D include trauma, neoplasms, and surgery [2]. However, AVP-D is also a frequent complication of hematologic disorders such as histiocytosis. In Langerhans cell histiocytosis (LCH), AVP-D affects up to 25–40% of patients while in Erdheim-Chester disease (ECD), it occurs in 25–48% of cases. In both conditions, AVP-D symptoms can precede the systemic manifestations of the disease by several years [3–6].

The clinical spectrum of systemic histiocytoses is broad and heterogeneous, often leading to diagnostic delay. Therefore, simple, noninvasive biomarkers capable of identifying early organ involvement, particularly neuroendocrine.

manifestations, are urgently needed. Copeptin is a recently identified biomarker for the diagnosis of AVP-D, and has proven useful in clinical practice due to its high sensitivity and specificity [7]. Copeptin is the C-terminal peptide of provasopressin and is co-secreted with AVP; the latter is short-lived in the bloodstream, whereas copeptin is stable for days and its plasma levels correlate with those of AVP; therefore, it represents an indirect and more stable measure of AVP secretion [8]. 

At physiological sodium levels, there can be an overlap between copeptin values in healthy individuals and those with partial AVP-D, limiting the diagnostic utility of unstimulated measurements. Copeptin diagnostic accuracy is considerably superior following osmotic stimulation with hypertonic saline infusion that induces a state of hypernatremia: in this setting, healthy subjects mount an appropriate copeptin rise whereas patients with AVP-D show no adequate response. A level of stimulated plasma copeptin < 4.9 pmol/L confirms a diagnosis of AVP-D, while levels ≥ 4.9 pmol/L indicate primary polydipsia. Measuring copeptin after hypertonic saline infusion simplifies the diagnostic algorithm while improving diagnostic accuracy compared to the traditional tests such as water deprivation [7–8].

Therefore, to establish AVP-D diagnosis in our cases (all but one), an osmotic stimulation test was performed via hypertonic saline infusion according to the guideline protocol [7]: a loading bolus of 250 mL of 3% NaCl was administered over 15–20 min, followed by a weight-adjusted continuous infusion of 0.15 mL/kg/min which was terminated when reaching a target serum sodium level > 149 mEq/L. Post-stimulation plasma copeptin concentrations were quantified via homogeneous time-resolved amplified cryptate emission (TRACE) technology utilizing the automated B·R·A·H·M·S KRYPTOR compact PLUS platform.

Several studies have demonstrated the utility of testing copeptin in common forms of AVP-D, such as those due to neurosurgery [9], however, its applicability in histiocytoses is yet to be determined. Herein, we report three cases of different forms of histiocytoses in which copeptin aided in the diagnosis of AVP-D. To the best of our knowledge, data on copeptin use in histiocytosis-related AVP-D are lacking.

### Case 1

**Fig. 1:**
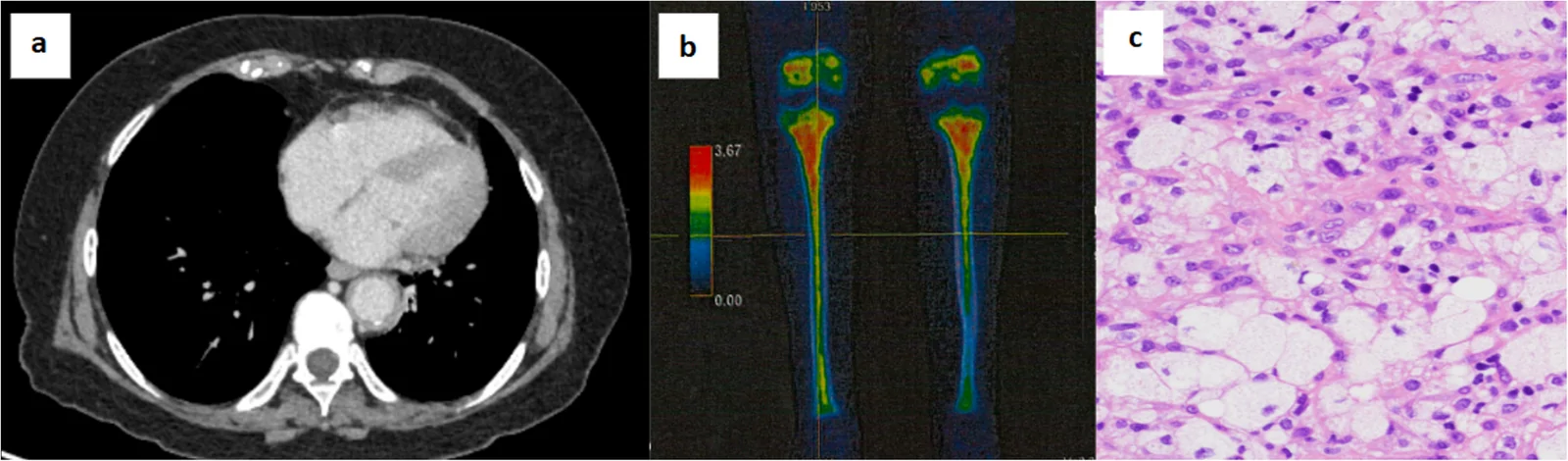
Imaging and biopsy findings of patient 1, affected by Erdheim-Chester disease. A. Computed tomography (CT) scan showing “sleeve” thickening of the aorta (green arrow). B. Positron emission tomography (PET) scan with 18F-fluorodeoxyglucose (FDG) showing diffuse hyperaccumulation of the tracer in the tibiae, bilaterally. C. Tibial Biopsy in patient 1: A hematoxylin-and-eosin staining (original magnification, 20X) shows diffuse infiltration by large foamy histiocytes, consistent with the diagnosis of Erdheim-Chester disease

**Fig. 2:**
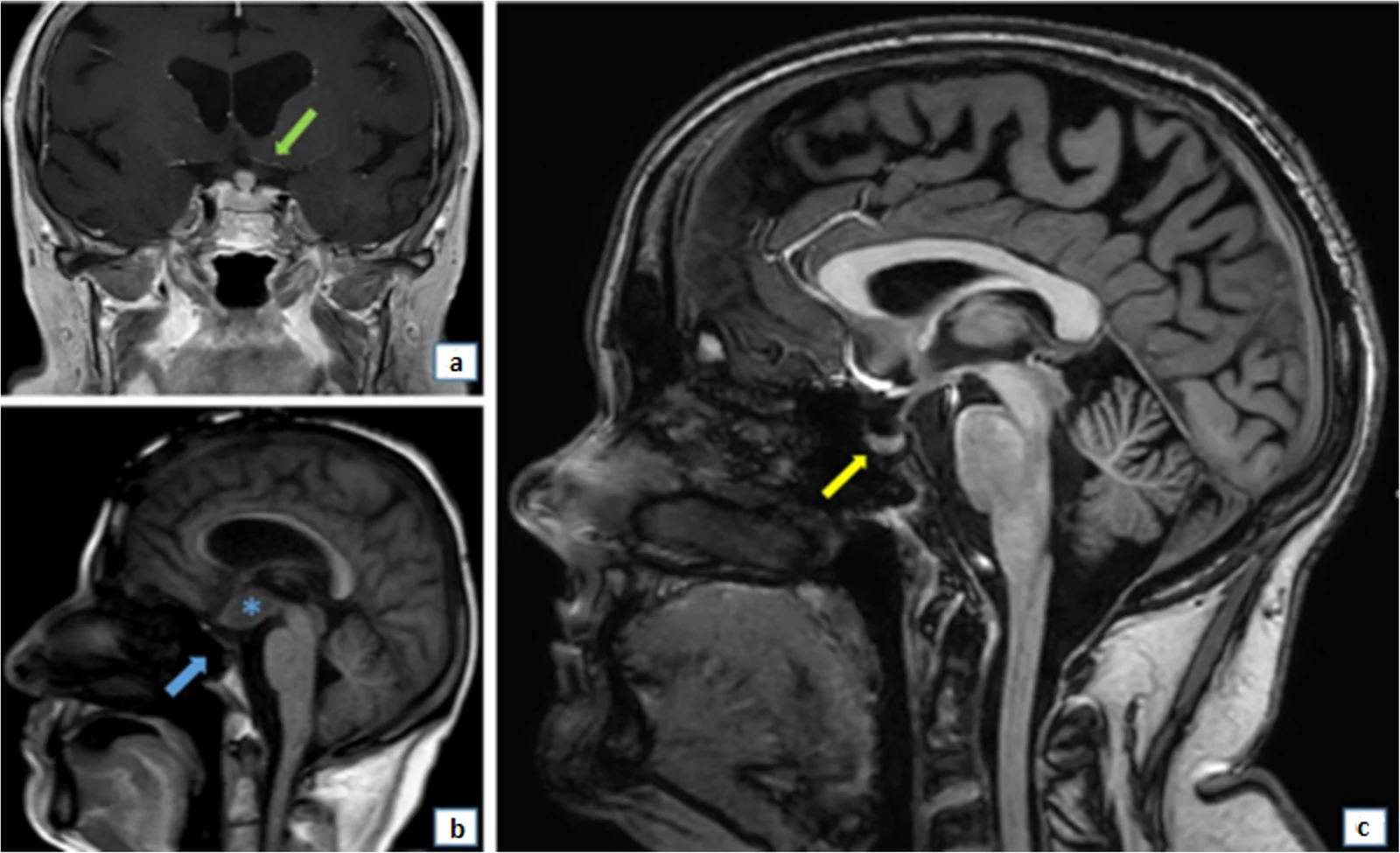
Pituitary radiological features of the three reported patients. A. Case 1: Erdheim-Chester disease. Contrast-enhanced coronal T1-weighted magnetic resonance imaging showing thickening of the pituitary stalk (green arrow); B. Case 2: Rosai-Dorfman-Destombes disease. Sagittal T1-weighted magnetic resonance imaging showing an empty sella (light blue arrow) and the presence of a hypothalamic lesion (light blue asterisk) C. Case 3: Langerhans cell histiocytosis. Sagittal T1-weighted magnetic resonance image showing a small pituitary gland and the absence of the normal hyperintense posterior pituitary “bright spot” (yellow arrow)

A 68-year-old woman presented with a polyuria-polydipsia syndrome, but no further endocrine evaluation was initially performed. Six months later, she started to complain of back pain and low-grade fever, and a magnetic resonance imaging (MRI) of the lumbosacral spine showed infiltration of the paravertebral soft tissues at the level of L4-L5. She also underwent a thoracoabdominal computed tomography (CT) scan, revealing circumferential periaortic infiltration (“coated aorta”) extending from the ascending aorta to the common iliac bifurcation, with avid contrast enhancement (Fig. 1A). Positron emission tomography (PET)-CT scan revealed multiple areas of fluorodeoxyglucose (FDG) uptake, including the posterior horn of the right lateral ventricle, the pituitary region, the sacroiliac region, the aorta and common iliac arteries, and the long bones of the lower limbs (Fig. 1B). The CT and PET-CT findings were suggestive of ECD, thus, a tibial biopsy was performed: histological examination revealed foamy histiocytic infiltration (Fig. 1C), typical of ECD, and molecular analysis demonstrated the *BRAF*V600E mutation. Brain MRI, performed to investigate the hypermetabolic lesions evidenced by PET-CT, showed thickening of the pituitary stalk (Fig. 2A): due to this finding, the polyuria/polydipsia syndrome was reconsidered and pituitary involvement suspected. The laboratory tests showed no deficits in the anterior pituitary function (normal thyrotropic, corticotropic and gonadotropic axes) but high plasma osmolality (296 mOsm/kg) with urinary hypo-osmolality (255 mOsm/kg). Given the strong suspicion of AVP-D, an osmotic stimulation test was performed and copeptin was 3.9 pmol/L, confirming the diagnosis. The patient started a targeted therapy with the BRAF inhibitor vemurafenib [10]. Given that polyuria (approximately 4 L/day) was adequately compensated by increased oral intake and serum sodium remained stable, desmopressin therapy was deferred. Three months after the initiation of vemurafenib, PET-CT demonstrated a complete metabolic response, without residual uptake in the previously affected areas. Accordingly, the patient showed a marked improvement in polyuria with a urine output of less than 2 L/day and normalisation of urinary and plasma osmolality.

### Case 2

**Fig. 3:**
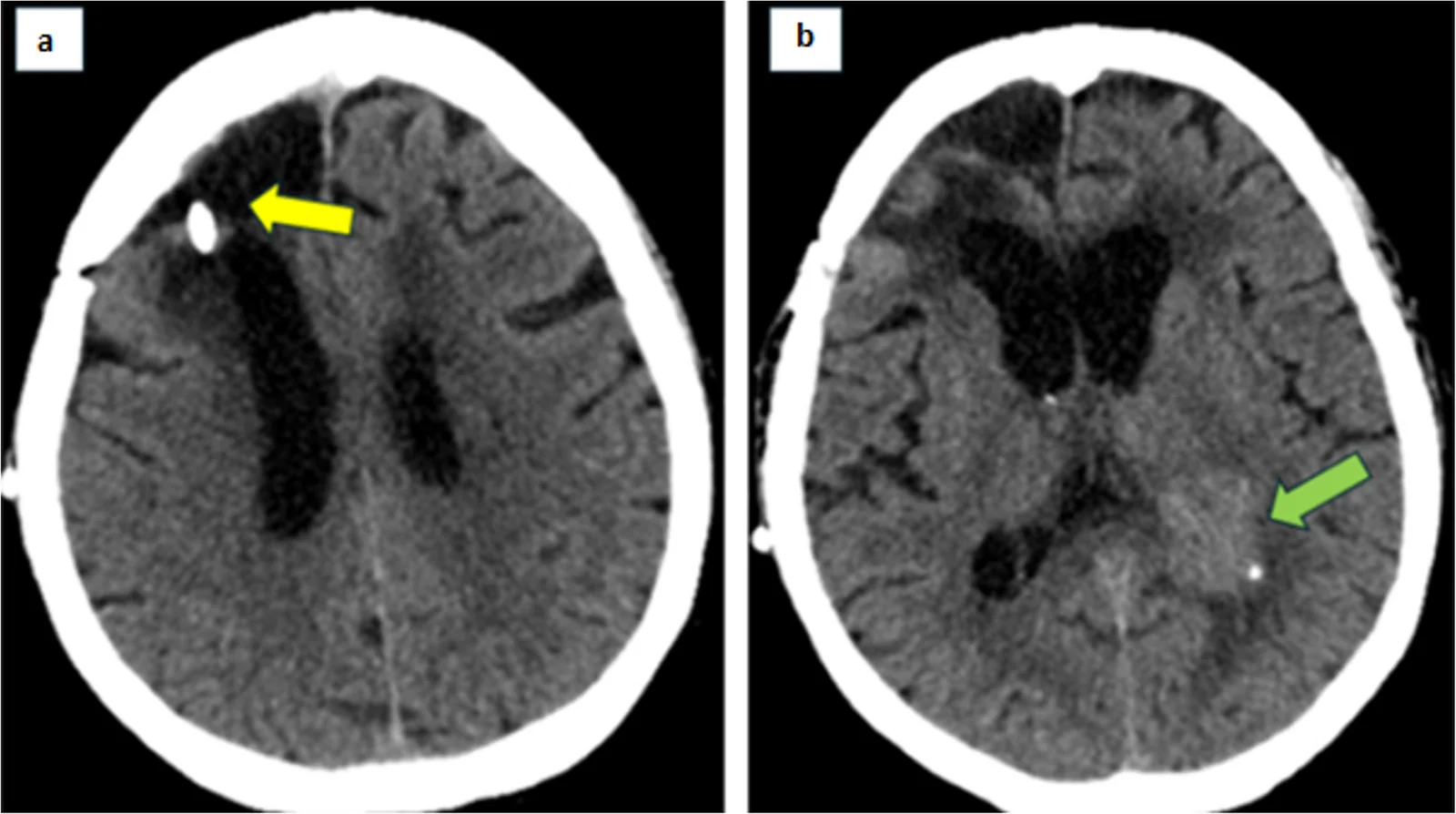
Brain involvement in patient 2, affected by Rosai Dorfman Disease. A. Axial computed tomography (CT) image showing postoperative changes, after right frontoparietal craniotomy and circular craniectomy of the operculum; the upper extremity of a ventriculo-peritoneal tube is seen (yellow arrow). B. Axial computed tomography (CT) imaging showing a mildly hyperdense expansive lesion involving the left posterior thalamic and parietal region, with compression of the adjacent ventricular trigone (light green arrow)

A previously healthy 60-year-old woman presented with new-onset confusion, urinary incontinence, and headache. CT and brain MRI revealed a right frontal hyperintense lesion, which required surgical resection. The histopathological examination was consistent with a diagnosis of meningocerebral Rosai-Dorfman-Destombes disease (RDD), and the follow-up brain MRI showed no evidence of residual disease. One year later the patient presented with a cognitive decline; a follow-up CT showed a possible multifocal recurrence of the intracranial disease (Fig. 3A, B); these lesions were FDG-avid on PET. Brain MRI confirmed the above findings and also showed an empty sella (Figure. 2B). Laboratory studies demonstrated progressively worsening hypernatremia (up to 162 mmol/L) and marked polyuria with low urine osmolality (110 mOsm/kg) and high plasma osmolality (336 mOsm/kg). Anterior pituitary evaluation revealed secondary hypothyroidism which required levothyroxine replacement therapy.

Considering the progressive polyuria and the MRI lesions (Fig. 2B), AVP-D was suspected [11]. Since the patient presented with a serum sodium of 162 mmol/L, a stimulation test with hypertonic saline was unnecessary and contraindicated. Despite this Na^+^ concentration, copeptin level was found to be profoundly suppressed at 3.3 pmol/L, confirming the diagnosis of AVP-D [11].

Treatment with desmopressin was promptly initiated and the patient exhibited a rapid and marked clinical and laboratory response, with a decline in serum sodium levels. In addition, due to the overall progression of the disease, treatment with cladribine in combination with glucocorticoids was also started.

### Case 3

A 16-year-old girl gradually developed polyuria and polydipsia and frequent episodes of hypotension (lipothymia). These clinical manifestations were, however, not severe and the patient did not seek medical advice. Fourteen years later, at the age of 30 years, she developed multiple cutaneous lesions. At the age of 36 years, a cutaneous biopsy was performed, leading to the diagnosis of LCH. Given this histological diagnosis, the history of polyuria-polydypsia was reconsidered. A brain MRI was performed: this revealed a small sellar cavity and the absence of the physiological posterior pituitary hyperintensity, consistent with involvement of the pituitary region (Fig. 2C). Laboratory evaluation revealed a serum sodium of 143 mEq/L, plasma osmolality of 296 mOsm/kg, and urine osmolality of 145 mOsm/kg. Anterior pituitary function was intact. Copeptin levels were measured after a hypertonic saline stimulation test, yielding a value of 2.0 pmol/L, consistent with the diagnosis of AVP-D. The patient was started on a MEKinhibitor (Trametinib) for LCH [12, 13], and also received hormone replacement therapy with desmopressin; this led to resolution of polyuria and polydipsia along with the lipothymic episodes and the normalization of plasma and urine osmolality. The patient also showed remission of cutaneous LCH lesions.

In Case 1, polyuria was the first manifestation of the disease, occurring months before overt disease onset, as reported in more than 10% of patients with ECD. In this case, an earlier use of copeptin (which proved to be low at the time of systemic disease presentation) could have helped to detect AVP-D and consequently facilitated the diagnosis of the systemic disease. In Case 2, in the setting of an acute event in a known histiocytosis (RDD), copeptin allowed the diagnosis of AVP-D even in a challenging clinical scenario, enabling prompt initiation of treatment, and preventing irreversible complications. Finally, in Case 3, the importance of establishing an accurate diagnosis of AVP-D from the pediatric onset of symptoms is highlighted, allowing further investigation of its underlying causes and ultimately leading to the identification of a rare disease such as LCH [12, 13]. 

## Discussion

Polyuria–polydipsia syndrome represents a diagnostic challenge. For decades, the diagnostic gold standard has been the water deprivation test, followed by the administration of desmopressin, a synthetic analogue of AVP, to assess the renal response to vasopressin. Performing these tests has historically been challenging, especially in complex patients such as those with rare infiltrative diseases. Our report supports the feasibility and clinical utility of copeptin measurement in. histiocytosis-associated AVP-D, particularly in complex scenarios where traditional testing is impractical or delayed [14]. 

It should be noted that copeptin is not a biomarker of histiocytosis, but rather a physiological surrogate of AVP secretion; its clinical value in this context lies in its ability to identify AVP-D, which represents one of the earliest and most frequent endocrine manifestations of histiocytic disorders. Brain MRI findings, including pituitary stalk thickening and loss of the posterior pituitary bright spot, are neither universally present nor predictive of clinical disease severity, and structural imaging alone cannot reliably reflect the functional integrity of the neurohypophysis [15]. MRI and copeptin should therefore be considered as complementary modalities: the first one provides structural information, while the latter offers a direct quantitative measure of residual vasopressin secretory capacity [8, 16]. 

Patients with apparently idiopathic AVP-D require prolonged follow-up, as systemic histiocytosis may manifest years after the initial presentation. Baseline evaluation should include a thorough assessment for signs and symptoms of systemic involvement (bone, skin, pulmonary, neurological or vascular), dedicated hypothalamic-pituitary MRI to identify infiltrative findings, and, in selected cases, FDG-PET/CT to detect extracranial disease sites, and eventually a biopsy for molecular characterisation with BRAF and MAPK pathway mutation testing. In addition, histiocytosis specialists should incorporate routine endocrine surveillance into the management of known histiocytosis, remaining vigilant for the onset of polyuria and polydipsia and educating patients to promptly recognise these symptoms.

While our series mostly featured isolated posterior involvement (except for Case 2), systematic anterior hormone screening is also required for long-term management of histiocytosis patients. In fact, anterior pituitary involvement is widely described in LCH (~ 20% of all patients), the most common manifestation being central hypogonadism, followed by hypothyroidism and hypocortisolism [2]. In ECD, anterior pituitary dysfunction is identified in up to 91.3% of patients, with growth hormone deficiency being the most prevalent deficit, followed by hyperprolactinemia, gonadotropic, thyrotropic, and corticotropic deficiency [6]. 

This study has limitations. The small number of cases and the retrospective nature of the observations preclude definitive conclusions regarding diagnostic accuracy. Furthermore, copeptin thresholds were extrapolated from studies conducted in other clinical contexts. Larger prospective studies are needed to validate optimal cut-off values in histiocytosis-related AVP-D. To conclude, early integration of copeptin into the diagnostic workup of systemic histiocytoses can facilitate prompt and accurate diagnosis of AVP-D, allowing initiation of potentially life-saving therapies.

## Data Availability

No datasets were generated or analysed during the current study.
